# Reutilization of solid wastes to improve the hydromechanical and mechanical behaviors of soils — a state-of-the-art review

**DOI:** 10.1186/s42834-023-00179-6

**Published:** 2023-05-16

**Authors:** Chih-Hsuan Liu, Ching Hung

**Affiliations:** Department of Civil Engineering, National Cheng Kung University, Tainan, 70101 Taiwan

**Keywords:** Solid waste, Geo-environment, Reutilization, Stabilized and reinforced soil (SRS), Containment, Optimum moisture content (OMC)

## Abstract

The rapid urbanization, industrialization, and population growth have led to a considerable rise in solid waste production, highlighting the need for efficient solid waste management and recycling methods. To address the challenge of solid waste production, an alternative solution is to repurpose it in geotechnical engineering. This offers promising benefits as solid waste exhibits various mechanisms that can improve soil's hydromechanical and mechanical behaviors. This review aims to comprehensively analyze the effects and potential application of various solid waste types to stabilize and reinforce soil. The impacts and research trends of industrial waste, such as fly ash, red mud, ground granulated blast-furnace slag, and construction and demolition waste, as well as agricultural and municipal solid wastes, including rice husk ash, press mud, used waste tires, and face masks, on soil properties were identified. The findings contribute to a better understanding of the potential of solid waste as a sustainable and cost-effective solution for improving soil quality, highlighting new research themes in this area. A wide range of innovative methods to stabilize and reinforce soil have also been proposed; however, ingenious and effective containment techniques, as well as addressing the potential impacts of climate change on stabilized and reinforced soils (SRS), still need to be developed for robust field applications. This state-of-the-art review offers useful insights into the reutilization of solid wastes as a promising alternative for improving the hydromechanical and mechanical behaviors of SRS.

## Introduction

Solid wastes are produced during various activities and are non-liquid or non-gaseous in nature. The increase in human population along with rapid urbanization and industrialization has created large amounts of waste. According to the World Bank [1], in 2016, the average amount of solid waste was approximately 0.74 kg cap^−1^ d^−1^ and the annual amount of solid waste was approximately 2010 Mt. Moreover, the latter is expected to reach 3400 Mt by 2025. This waste poses a risk to all living things, as well as the environment. Therefore, proper solid waste management/reutilization is necessary to mitigate the problems related to the generation of large accumulations of solid waste.

**Fig. 1:**
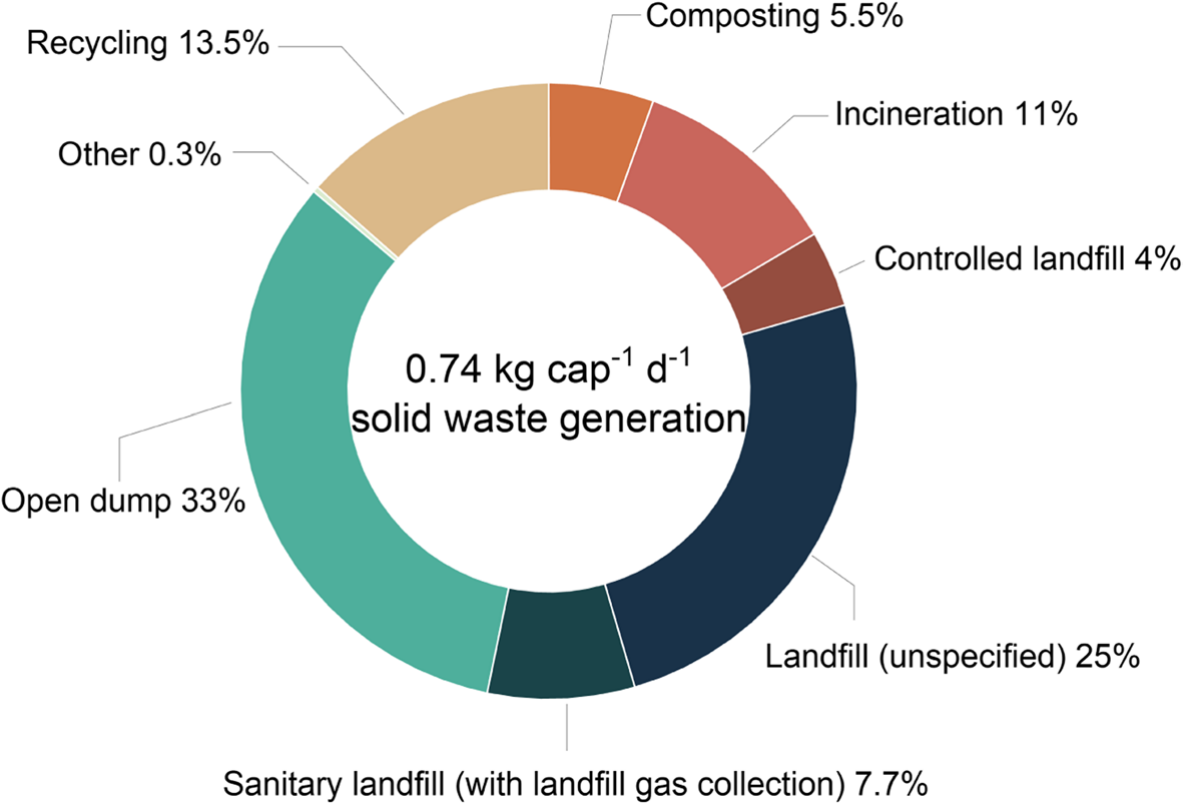
Solid waste management methods (Kaza et al. [1] with modifications, data in 2018)

Waste management aims to provide sanitary living conditions by reducing the amount of material entering or leaving society and encouraging material reutilization within society [2]. The general processes of waste management are (a) the collection, handling, and transport of wastes and (b) the processing, disposal or recycling, and treatment of wastes [2, 3]. Typical methods for managing solid waste include composting, incineration, recycling, and disposal in landfills or open dumps. Figure 1 shows that approximately 40, 19, and 11% of waste is disposed in landfills, recycled and composted, and incinerated, respectively. Because the amount of waste generated, waste composition and income vary among countries, considerable research has been conducted to investigate appropriate solid waste management practices in different countries to minimize environmental pollution and maximize resource recovery [4–10].

Numerous researchers have recently demonstrated that using solid wastes in geotechnical engineering, especially in stabilized and reinforced soils (SRS), is a suitable solid waste management method. SRS results have been reported by adding various types of additives, including chemicals [11–16], biochemicals [17–19], fibers [20–22], and solid wastes, to improve their weak engineering properties. Traditional stabilizing agents may have negative impacts on the environment and ecology [23, 24]; for example, the production of cement or lime consumes high amounts of energy and can result in carbon dioxide (CO_2_), methane (CH_4_), and nitrous oxide (N_2_O) emission [25, 26]. Accordingly, different researchers have explored the use of solid wastes as an alternative material to ensure that the geotechnical properties of problem soils meet the minimum standard requirement [27–36].

Despite the effectiveness of many waste materials in enhancing soil behaviors, the use of various waste materials for forming SRS and their soil improvement mechanisms have yet to be fully understood. The objective of this study is to provide a comprehensive review of various solid wastes that have been widely studied or applied in the context of SRS, including their mechanisms and remarks related to soil improvement and hydromechanical and mechanical properties of SRS. The state-of-the-art review will serve as a valuable guide for researchers and practitioners in the field of SRS, while also highlighting areas that require further investigation.

## Solid wastes used for forming SRS

This section presents the solid wastes that have been used as stabilizers and that can improve the hydromechanical and mechanical properties of problem soils, including fly ash (FA), red mud (RM), ground granulated blast-furnace slag (GGBS), construction and demolition (C&D) waste, rice husk ash (RHA), press mud (PM), waste tire, and face mask (FM). The solid wastes are classified into (a) industrial, (b) agricultural, and (c) municipal solid waste, as listed in Table 1. The effects of each solid waste on the hydromechanical and mechanical properties of problem soils are presented, and the problems and shortcomings in the application of each type of solid waste as a soil stabilizer are discussed.

**Table 1:** Classification of solid waste

Classification	Solid waste
Industrial solid waste	FA; RM; GGBS; C&D waste
Agricultural solid waste	RHA; PM
Municipal solid waste	Waste tire; FM

### Industrial solid wastes used for forming SRS

Industrial solid waste refers to waste generated from industrial activities, including any solid material that becomes useless during the manufacturing process [37]. Some of the research related to using industrial solid wastes for forming SRS are described in the following sections.

#### FA

**Fig. 2:**
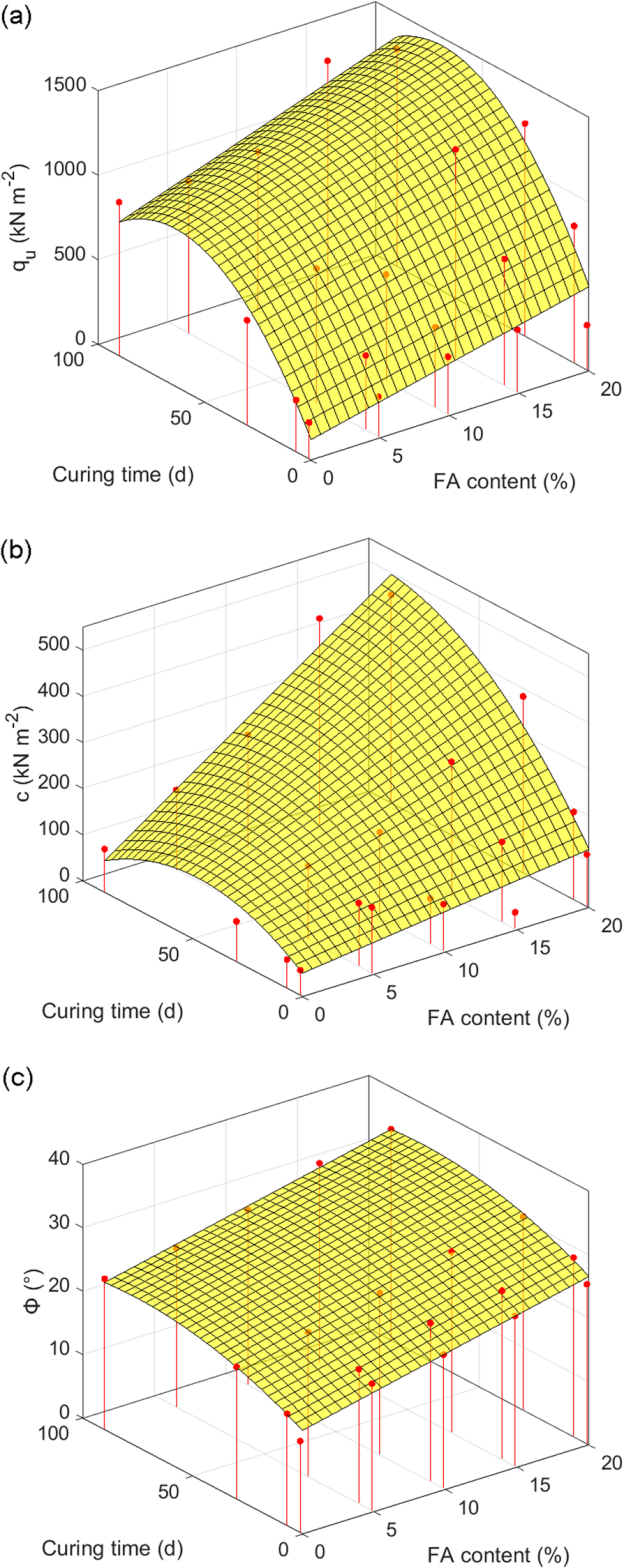
Relationship among curing time, FA content, and (a) *q*_*u*_; (b) *c*; (c) *ϕ* of FA-treated clayey soil

FA is a by-product of the burning of pulverized coal in thermal power plants [38, 39]. According to a survey [40], approximately 780 Mt of FA are produced globally each year. The reutilization of FA has significant environmental and economic benefits because it contains SiO_2_, Al_2_O_3_, Fe_2_O_3_, and CaO, which have potential pozzolanic reactivity [41], low unit weight and compressibility [28], and are cost-effective and energy-efficient [28]. FA can be divided into Class C and Class F according to the type of coal burned, corresponding to high-lime and low-lime FAs, respectively [27, 42]. The use of FA as pozzolanic material to replace cement has been extensively investigated. For instance, Sezer et al. [27] investigated the effects of high-lime FA (Class C) on the strength characteristics of a soft clay subgrade of a military zone in Izmir, Turkey. The results showed that the inclusion of FA improved the soil properties, including unconfined compressive strength, *q*_*u*_, and shear strength parameters (cohesion, *c,* and internal friction angle, *ϕ*), and the effects of FA increased with the increase in curing time (t) and content (Fig. 2). The improvement in soil properties after treatment with FA is attributed to the pozzolanic reaction and pore refinement effect of FA, as well as its high free-lime content. In addition, multiple regression models for predicting the *q*_*u*_, *c*, and *ϕ* of the FA-treated soil, as shown in Eqs. (1, 2, 3), respectively, have been developed.

1 $$\mathrm{q}_{u}(\mathrm{kN m}^{-2}) = 247.1034 + 22.2932 \times \mathrm{ FA}(\mathrm{\%}) + 7.5042 \times \mathrm{ t }(\mathrm{d})$$

2 $$\mathrm{c }(\mathrm{kN m}^{-2}) = 20.7375 + 9.9473 \times \mathrm{ FA}(\mathrm{\%}) + 11.9468 \times \mathrm{ t }(\mathrm{d})$$

3 $$\upphi (^\circ ) = 17.3267 + 0.5083 \times \mathrm{ FA}(\mathrm{\%}) + 0.0698 \times \mathrm{ t }(\mathrm{d})$$

The above-mentioned study confirms that FA can improve soil characteristics. Additionally, some researchers have found that incorporating an activator or secondary additive can further enhance the soil-stabilization effects of FA [29, 30, 43]. For example, Karami et al. [29] conducted a series of mechanical tests, including California bearing ratio (CBR), standard Proctor compaction, aided with various microscopic tests, including scanning electron microscope (SEM), thermogravimetric analysis (TGA), Fourier transform infrared (FT-IR), and X-ray diffraction (XRD) tests, which help identify the mechanisms involved in soil stabilization, to evaluate of the role of secondary additives, such as lime, calcium sulfoaluminate (CSA) cement, enzymes, and polymers in improving Class F FA-treated soil. Their results revealed that adding secondary additives can further enhance the strength of FA-treated soil, with the addition of lime and enzymes to FA-treated soil producing the best performance (highest CBR value). This may be due to the combined effect of the formation of pozzolanic and cementitious products and density enhancement. Accordingly, it can be concluded that Class C FA, owing to its high lime content, might have a better reaction with soil, resulting in more effective stabilization. Therefore, Class C FA presents an opportunity for applications where there is no need for secondary additives, thereby offering a more cost-effective alternative for a wide range of stabilization applications. The various studies on FA used for forming SRS are summarized in Table 2.

**Table 2:** Use of FA for forming SRS

**Reference**	**Soil**	**Treatment**	**Treatment content**	**Tests**	**Effects of treatment**	**Primary mechanism**	**Remarks**^a^
Sezer et al. [27]	Soft clay subgrade	High-lime FA (Class C)	0–20%	Compaction; UCS; direct shear	Increases optimum moisture content (OMC), UCS, cohesion, and friction angle and decreases maximum dry density (MDD)	Cementation of hydration products and pore filling	The level of improvement (increases OMC, UCS, cohesion, and friction angle, and decreases MDD) increases with an increase in FA content
Karami et al. [29]	Expansive clay soil	Class F FA; Secondary additives (CSA cement; enzyme; polymers)	7.5 and 15% (FA); 3% (CSA cement); dilution mass ratio of 1:500 and application mass ratios of 3% (enzyme); 3% (polymer)	CBR; compaction; SEM; XRD; TGA; FT-IR	Secondary additives can be effectively used to improve the efficiency of FA based soil stabilization	Cementation of hydration products and density enhancement	Adding lime and enzyme to FA-treated soil produced the best performance (highest CBR)
Tastan et al. [44]	Organic soil	Classes C and F FA	10–30%	UCS; resilient modulus	Increase UCS and resilient modulus	Cementation of hydration products	The highest UCS and resilient modulus were obtained when the CaO content of FA was greater than 10% and CaO/SiO_2_ ratio of FA was 0.5–0.8

^a^Under the studied conditions

#### RM

RM is the reddish–brown alkaline by-product of bauxite processing during alumina production. Depending on the grade of raw bauxite and the type of alumina extraction process, RM can be divided into Bayer RM, sintering RM, and combined process RM, of which more than 95% is Bayer RM [45–49]. An estimated 120 Mt of RM are produced globally each year [50]. Due to the strong alkalinity and hydrolysis of RM, the addition of RM to the soil as a partial alternative to cementitious material (e.g., cement and lime) can boost the pozzolanic, carbonization, and ion exchange reactions to generate more cementitious gels, such as hydrated calcium silicate (C–S–H), hydrated calcium aluminate (C–A–H), hydrated calcium silico–aluminate (C–A–S–H), and ettringite [51], leading to a denser structure. The pozzolanic mechanism can be explained by reactions, as shown in Eqs. (4, 5, 6):

4 $${\mathrm{Ca}}^{2+}+2\left(\mathrm{OH}\right)^{-}+{\mathrm{SiO}}_{2 }\to \mathrm{C}-\mathrm{S}-\mathrm{H}$$

5 $${\mathrm{Ca}}^{2+}+2\left(\mathrm{OH}\right)^{-}+ {\mathrm{Al}}_{2}{\mathrm{O}}_{3}\to \mathrm{C}-\mathrm{A}-\mathrm{H}$$

6 $${\mathrm{Ca}}^{2+}+2\left(\mathrm{OH}\right)^{- }+{\mathrm{SiO}}_{2 }+{\mathrm{Al}}_{2 }{\mathrm{O}}_{3} \to \mathrm{C}-\mathrm{A}-\mathrm{S}-\mathrm{H}$$

Chen et al. [31] evaluated the dynamic characteristics and environmental impact (dynamic stress, *σ*_*d*_, moisture content, *w*, confining pressure, *σ*_*3*_, and loading frequency, *f*) of loess treated with a combination of RM and a small amount of cement (RM-cement) under adverse conditions. The results demonstrated that *σ*_*d*_ and *w* have a greater effect on treated loess than *σ*_*3*_ and *f*. The higher *w* significantly reduced the dynamic load resistance of the treated loess; however, the addition of RM-cement can still improve the microstructure and water sensitivity of the loess. The enhancement in the loess dynamic characteristic via the addition of RM-cement can be attributed to the pore filling effect, the dissolution of active ions, and strong alkalinity, which promote the pozzolanic reaction to produce more gelation. Wan et al. [32] conducted a series of experimental tests, including unconfined compressive strength (UCS), hydraulic conductivity, and microscopic tests to investigate potential use of combination of RM and phosphogypsum (PG) in improving cement-treated marine dredged clay (MDC). MDC mixed with 5% RM and 5% PG exhibited higher UCS, which is attributable to the alkaline environment and Ca^2+^ produced by RM and PG, respectively, significantly increasing the early strength. In addition, SO_4_^2−^ produced by PG promotes the breaking of Si–O and Al–O bonds in the RM and MDC in an alkaline environment, resulting in the generation of more cementitious gels (C–S–H, C–A–H, C–A–S–H, and ettringite). The studies on the use of RM for forming SRS are summarized in Table 3.

**Table 3:** Use of RM for forming SRS

Reference	Soil	Treatment	Treatment content	Tests	Effects of treatment	Primary mechanisms	Remarks^a^
Chen et al. [51]	Loess	RM; cement	0–25% (RM); 5% (cement)	Compaction; UCS; resistivity; dynamic triaxial; microscopic	Increases compaction, strength, and dynamic properties	Cementation of hydration products and pore filling	The optimum RM content is approximately 15%–20%
Chen et al. [31]	Loess	RM; cement	15% (RM); 5% (cement)	Dynamic triaxial; leaching toxicity; microscopic	Increases failure dynamic stress, and dynamic elastic modulus	Cementation of hydration products and pore filling	-
Wan et al. [32]	MDC	RM; PG	0–10% (RM and PG)	UCS; hydraulic conductivity; microscopic	Increases UCS and decreases hydraulic conductivity	Cementation of hydration products and pore filling	5% RM + 5% PG is most effective in improving UCS of MDC

^a^Under the studied conditions

#### GGBS

GGBS is a by-product of the iron manufacturing industry. During the manufacture of iron, iron oxides, coke, and limestone are fed into a blast furnace, where the iron oxides are reduced to iron and separated from the molten slag (known as blast-furnace slag). When the molten slag is rapidly quenched by granulation in water, it forms a fine, granular, mostly non-crystalline, glassy substance, namely, granulated blast-furnace slag. GGBS is then obtained by drying and grounding the granulated blast-furnace slag into a fine powder. According to research, the annual global production of GGBS is approximately 530 Mt [52]. GGBS is mainly composed of calcium, aluminum, silica, magnesium, and oxygen [53–55]. Because GGBS is a latently hydraulic material, it is usually blended with cement or activated by lime for soil stabilization [35, 36]. GGBS used alone exhibits a slow hydration rate and, therefore, a very low early strength. Thus, chemical activation is required to increase its hydration rate and produce compounds such as C–S–H, C–A–H, or C–A–S–H [33, 34]. Many chemicals can be used as GGBS activators, among which NaOH, Na_2_CO_3_, Na_2_O·nSiO_2_ (water glass), CaO, and Na_2_SO_4_ are the most widely used and economical [34]. Lime-GGBS has been used for soil stabilization for more than 10 years, most of which involve the suppression of expansion associated with sulfates or sulfides in lime-treated soil incorporating GGBS [33, 34, 36, 56].

Yi et al. [36] used quick lime and hydrated lime to activate GGBS for marine soft clay stabilization and compared the result to the use of cement. The results demonstrated that both types of lime-activated GGBS-treated marine soft clays achieved a higher UCS than cement-treated soil when the lime/GGBS ratio (by dry weight) was larger than 0.1. The quick lime-activated GGBS-treated marine soft clay cured for 7 and 28 d yielded slightly higher UCS than hydrated lime-activated GGBS-treated marine soft clay, whereas opposite results were observed in the sample cured for 90 d. This may be because that, under the content, quick lime is hydrated to become more hydrated lime, which improves the early-age GGBS-activating effect and reduces the water content of marine soft clay. On the other hand, the binding capacity of C–S–H decreases with increasing Ca/Si ratio, resulting in a relatively low UCS in marine soft clay cured for 90 d. Because the weight of quick lime increases by 1.32 times after hydration to form hydrated lime, a relatively lower UCS can be achieved in the marine soft clay treated with quick lime-activated GGBS.

Reactive MgO has recently been determined to be more efficient than Ca(OH)_2_ in activating GGBS, resulting in a higher soil strength development rate. A series of tests, including UCS, permeability, and nondestructive analyses, were conducted by Yi et al. [33] to compare the improvement in MgO-activated and lime-activated GGBS-treated soil. The results showed that at optimum GGBS/MgO ratios of 19:1–4:1, varying with additive content and curing duration, MgO-activated GGBS-treated soil yielded higher UCS than lime-activated GGBS-treated soil. In MgO-activated GGBS-treated soil, hydrotalcite, in addition to hydration products similar to those of cement, was produced. The studies on GGBS used for forming SRS are summarized in Table 4.

**Table 4:** Use of GGBS for forming SRS

Reference	Soil	Treatment	Treatment content	Tests	Effects of treatment	Primary mechanism	Remarks^a^
Singhi et al. [53]	Clayey soil	GGBS; FA; alkali activators	4–20% (GGBS/ FA or combination both)	UCS	Increases UCS	Cementation of hydration products	UCS increases with the increase in GGBS and FA content
Yi et al. [35]	Sand; Clayey silt	GGBS; activators (reactive magnesia, brucite, and hydrated lime)	5% and 10% (GGBS for the sand); 10% and 20% (GGBS for the clayey silt); 0.05–0.40 (activators/GGBS)	UCS; XRD; SEM	Increases UCS	Cementation of hydration products	Reactive magnesia yields the highest activating efficiency
Sharma and Sivapullauah [54]	Expansive soil	GGBS; FA	70:30 (GGBS: FA); 10–40% (GGBS-FA mixture)	Atterberg limit; UCS; SEM; XRD	Increases UCS and MDD and decreases swelling potential, liquid and plastic limits, and OMC	Cementation of hydration products	The optimum GGBS-FA content was 20%
Yi et al. [34]	Marine soft clay	GGBS; activators (NaOH, Na_2_CO_3_, and Na_2_SO_4_); carbide slag; cement	30% (GGBS and cement); 0.1 (Carbide slag/GGBS)	UCS; XRD; SEM; MIP	Enhances the strength development rate	Cementation of hydration products	Na_2_SO_4_-carbide slag-GGBS was found to be the optimum binder
Yi et al. [36]	Marine clay	GGBS; activators (quick lime and hydrated lime)	20% (GGBS); 0.05–0.40 (Quick lime and hydrated lime/GGBS)	UCS; XRD; SEM; MIP	Increases UCS	Cementation of hydration products	The optimum lime/GGBS ratio was 0.2 at 7 and 28 curing days, and 0.10 at 90 curing days

^a^Under the studied conditions

#### C&D waste

**Fig. 3:**
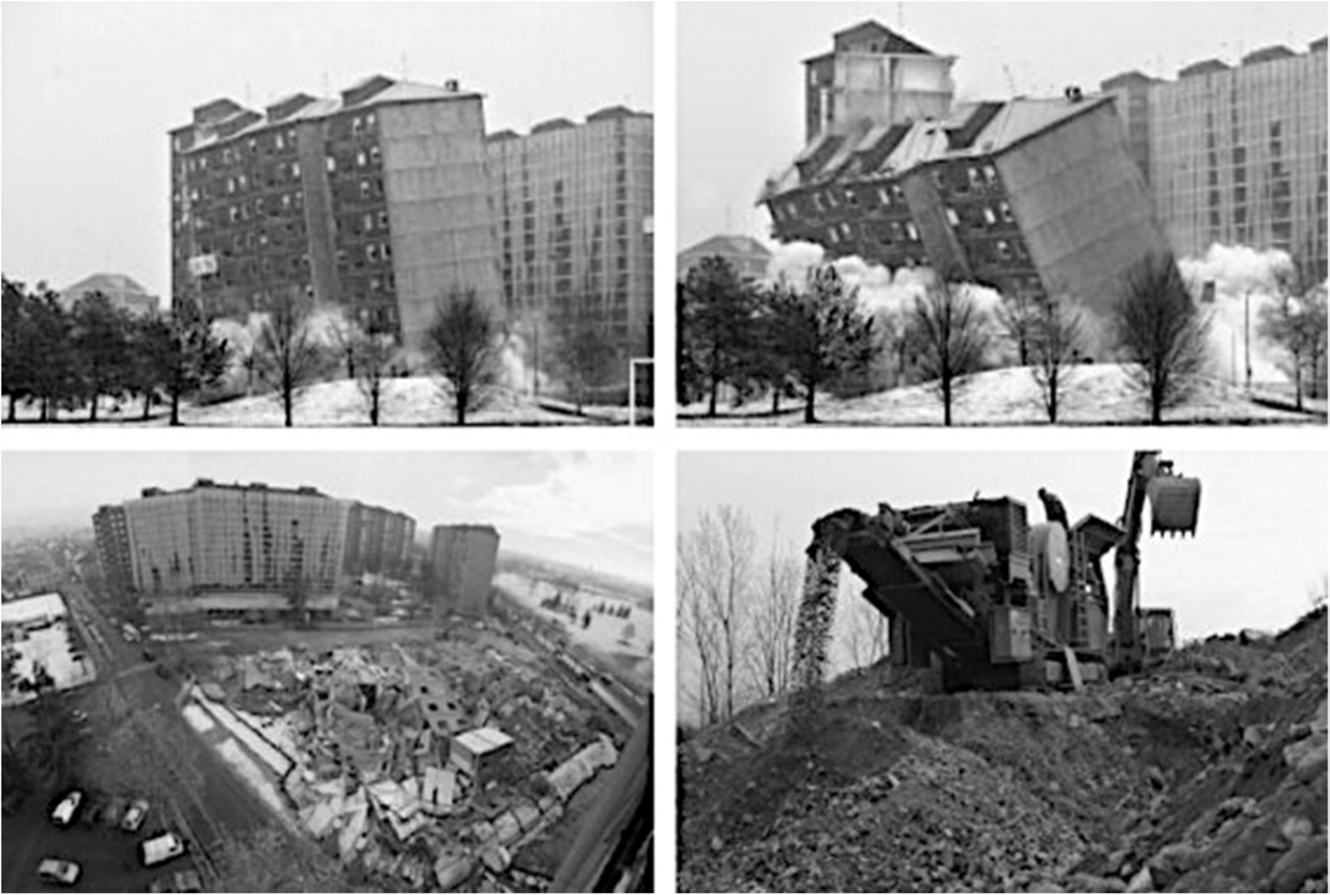
Blast demolition and rubber recycling of the building (adopted from Blengini [61])

According to the United States Environmental Protection Agency [57], C&D waste refers to waste generated when new building and civil engineering infrastructures are constructed by demolishing or renovating existing buildings or civil engineering infrastructures, including concrete, brick, reclaimed asphalt, steel, timber, plastics, and other building materials and products (Fig. 3). The rapid growth of the infrastructure sector has resulted in the generation of large amounts of construction waste globally [58]. This waste is mostly dumped on already scarce landfill sites, causing land, resource, and material depletion and deterioration [59, 60]. Due to the increasing scarcity of natural resources and the growing cost of disposal in landfills in many countries, researchers around the world have been driven to seek more sustainable solutions to these problems and reuse waste materials. In particular, the use of recycled C&D waste in geotechnical engineering applications has gained popularity.

Recycled concrete aggregate (RCA), crushed brick (CB), waste rock (WR), reclaimed asphalt pavement (RAP), and fine recycled glass (FRG) are the five predominant types of C&D wastes. In addition to being used as an additive to improve soil performance, C&D waste has been utilized alone as a geomaterial in applications such as pavement subbase or as fill material for geosynthetic reinforced infrastructures. Therefore, their geotechnical properties are studied by various researchers as presented in Table 5 [62]. Because C&D waste is pozzolanic, it has the potential for soil stabilization [63]. Sharma and Hymavathi [63] investigated the effects of FA, pulverized C&D waste, and lime use on the geotechnical characteristics of clayey soil via a number of tests, including differential free swell, pH, compaction, UCS, and CBR tests. The C&D waste used in the study is mainly composed of a cement concrete layer covered by a cement-sand-rich mortar layer with tiling above. The results revealed that the addition of C&D waste decreased differential free swell and maximized dry density but increased pH, UCS, and soaked CBR. Such an improvement can be attributed to the pozzolanic reaction between soil and C&D waste. Cristelo et al. [64] investigated the stabilization of pulverized C&D waste with a high fine content using alkali-activated FA. The mechanical behavior was assessed through UCS tests, and the reaction products were characterized via XRD, FT-IR, SEM, and back-scattered electron microscopy (BSEM)/energy-dispersive X-ray spectroscopy. The elemental constitution of C&D waste allowed C–A–S–H gel formation in the early stage under alkaline activation, whereas the progressive addition of FA promoted the coexistence of C–A–S–H and N–A–S–H gels, especially after 4 weeks of curing. The formation of the gels improves the UCS results. Hasan et al. [65] investigated the effect of GGBS and C&D waste on bentonite clay stabilization through UCS tests and microanalysis tests (SEM, XRD, and energy dispersive spectrometer). The increase in UCS after the addition of GGBS and C&D waste, with long curing, was pronounced. This may be due to the formation of structural bonds between additives and bentonite in treated soil, considering slag crystals and bentonite particles were observed to occupy cavities and vesicles on C&D waste particles. Moreover, no significant improvement was observed if only C&D waste was added to the soil. The above literature suggests that C&D waste has a high potential for soil stabilization. The studies on the role of C&D waste used for forming SRS are summarized in Table 6.

**Table 5:** Geotechnical properties of C&D waste [62]

Geotechnical properties	RCA	CB	WR	RAP	FRG	Quarry material
*D*_*10*_ (mm)	0.24	0.18	0.075	0.24	0.16	-
*D*_*30*_ (mm)	1.3	1.7	1.5	1.9	0.45	-
*D*_*50*_ (mm)	5.0	5.6	3.9	4.5	0.85	-
*D*_*60*_ (mm)	7.5	8.0	5.6	5.9	1.2	-
*C*_*u*_	31.2	44.4	74.7	25.6	7.5	-
*C*_*c*_	0.9	2.0	5.4	2.5	1.5	-
Gravel content (%)	50.7	53.6	44.7	48.0	0.0	-
Sand content (%)	45.7	39.8	45.1	46.0	94.6	-
Fines content (%)	3.6	6.6	10.2	6.0	5.4	< 10
^**a**^USCS classification	GW	GW	SW	GW	SW	-
Particle density–coarse fraction (kN m^−3^)	27.1	26.2	28.1	23.5	24.4	> 19.62
Particle density–fine fraction (kN m^−3^)	26.0	25.8	28.0	23.4	24.3	> 19.62
Water absorption–coarse fraction (%)	4.7	6.2	3.3	2.2	1.0	< 10
Water absorption–fine fraction (%)	9.8	6.9	4.7	2.4	1.8	< 10
MDD (kN m^−3^)–modified compaction	19.13	19.73	21.71	19.98	17.40	> 17.5
OMC (%)–modified compaction	11.0	11.25	9.25	8.0	10.5	8–15
Organic content (%)	2.3	2.5	1.0	5.1	1.3	< 5
pH	11.5	9.1	10.9	7.6	9.9	7–12
Hydraulic conductivity (m s^−1^)	3.3 × 10^–8^	4.5 × 10^–9^	2.7 × 10^–7^	3.5 × 10^–7^	1.7 × 10^–5^	> 1 × 10^–9^
Flakiness index	11	14	19	23	-	< 35
Los Angeles abrasion loss (%)	28	36	21	42	25	< 40
California Bearing Ratio (%)	118–160	123–138	121–204	30–35	42–46	> 80
Triaxial test: apparent cohesion (kPa)	44	41	46	53	0	> 35
Triaxial test: friction angle (degree)	49	48	51	37	37	> 35
Resilient modulus: targeting 90% OMC	239–357	301–319	121–218	-	-	125–300
Resilient modulus: targeting 80% OMC	487–729	303–361	202–274	-	-	150–300
Resilient modulus: targeting 70% OMC	575–769	280–519	127–233	-	-	175–400

^a^Unified Soil Classification System (USCS) [66]

**Table 6:** Use of C&D waste for forming SRS

Reference	Soil	Treatment	Treatment content	Tests	Effects of treatment	Primary mechanism	Remarks^a^
Sharma and Sharma [58]	Clayey soil	RHA; C&D waste; lime	0–16% (RHA); 0–28% (C&D waste); 0–5% (lime)	Differential free swell; Compaction; UCS; CBR; resilient modulus	Increases UCS, CBR, and resilient modulus and decreases differential free swell	Cementation of hydration products	A combination of both the wastes and lime is best suited for the enhancement in UCS
Sharma and Hymavathi [63]	Clayey soil	FA; C&D waste; lime	0–22% (FA); 0–36% (C&D waste); 3–6% (lime)	Differential free swell; pH; compaction; UCS; CBR	Increases pH, UCS, and CBR and decreases differential free swell and MDD	Cementation of hydration products	For the studied clayey soil, lime is the best stabilizer to be used as subgrade material; early gain of strength is the primary importance: C&D waste; long-term strength is the primary criteria: FA
Hasan et al. [65]	Bentonite clay	GGBS; C&D waste	2–5% (GGBS); 10–20% (C&D waste)	UCS; microanalysis	Increases UCS	Cementation of hydration products	The optimum additive ratio was combination of 5% GGBS and 20% C&D waste
Sharma and Sharma [67]	Clayey soil	C&D waste	4–28%	Differential free swell; Atterberg limits; compaction; UCS; CBR; permeability	Increases UCS, CBR, permeability, and secant modulus and decreases MDD and OMC	Cementation of hydration products; coarser particle of C&D waste and higher coefficient of permeability compared to the clayey soil	The optimum C&D waste content was 24%
Sharma and Sharma [68]	Expansive clay	C&D waste	0–32%	Atterberg limits; pH; CD triaxial compression	Improves stress–strain and volumetric behavior of expansive clay; increases angle of shearing resistance, strength ratio, and stiffness and decreases volumetric strain and cohesion	Not discussed	The optimum C&D waste content was 24%

^a^Under the studied conditions

### Agricultural solid wastes used for forming SRS

Agricultural solid waste refers to waste from the growing and processing of raw agricultural products, such as fruits, vegetables, and other crops [69, 70]. The following sections of this review will focus on SRS based on RHA and PM.

#### RHA

Rice husk is an agricultural solid waste obtained from the outer cover of rice during milling. According to the Food and Agriculture Organization (FAO) [71], global rice production in 2020 was approximately 757 Mt. An estimated 23% of rice husk is from rice production [72], and approximately 174 Mt per year of rice husk is generated worldwide. Generally, rice husk is disposed of by dumping or burning in boilers. The RHA produced by burning rice husk is approximately 20% of its weight [73]. RHA reacts with CaO and SiO_2_ to produce C–S–H, which is the main parameter affecting SRS. Previous studies investigated the addition of RHA and other materials for soil stabilization, and the results have revealed that RHA is a promising secondary material for improving lime or cement-treated soil [74–76]. Basha et al. [74] evaluated the properties of residual soil treated with RHA, cement, and a combination thereof via a series of experimental tests, including Atterberg limits, compaction, UCS and durability, and CBR tests. The results showed that RHA can improve the properties (reduction in PI and increase in strength and resistance to immersion) of the residual soil either solely or mixed with cement. In general, the addition of 6–8% of cement and 10–15% RHA to the residual soil produced the optimum improvement in the soil properties.

To maximize the pozzolanic reactivity of RHA, some treatments such as grinding, acid/alkali, and calcium-based material excitation can be used to improve the physical and chemical properties of RHA. By grinding RHA to smaller particle size, surface energy, electrostatic Coulomb forces, and van der Waals forces between particles of the RHA can be improved, thereby enhancing the pozzolanic and filling effects of RHA [77]. Moreover, leaching rice husks with acetic acid and oxalic acid before burning them to RHA improved the purity, reactivity, brightness, surface area, and pore volume [78]. The studies on the use of RHA used for forming SRS are summarized in Table 7.

**Table 7:** Use of RHA for forming SRS

Reference	Soil	Treatment	Treatment content	Tests	Effects of treatment	Primary mechanism	Remarks^a^
Basha et al. [74]	Residual soil	RHA; cement	0–34% (RHA); 0–12% (cement)	Atterberg limits; compaction; UCS; durability; CBR	Increases OMC, UCS, CBR, and resistance and decreases MDD and plasticity	Cementation of hydration products	6–8% of cement and 15–20% RHA show the optimum improvement
Canakci et al. [79]	Expansive soil	RHA; rice husk powder; lignin	0–10% (RHA); 0–20% (rice husk powder and lignin)	Atterberg limits; compaction; swelling; UCS	Increases UCS and plastic limit and decreases liquid limit, plasticity index, and swelling percentage	Cementation of hydration products	With increasing RHA content, the UCS increases
Hossain [75]	Clayey soil	RHA; cement kiln dust	0–20% (RHA and cement kiln dust)	Compaction; UCS; CBR; Atterberg limits; splitting tensile strength; modulus of elasticity; durability	Increases compressive and tensile strengths, modulus of elasticity, CBR, and durability	Cementation of hydration products	The level of improvement increases with an increase in additive content
Sharma et al. [80]	Expansive soil	Lime; calcium chloride; RHA	0–5% (lime); 0–2% (calcium chloride); 0–16% (RHA)	UCS; CBR	Increases UCS and CBR	Cementation of hydration products	The optimum RHA content was 12%

^a^Under the studied conditions

#### PM

PM is one of the solid wastes left after sugarcane juice filtration in sugar industries and is also known as sugar filter mud or filter cake [81]. According to FAO [71], the global annual production of sugarcane reached 1870 Mt in 2020. PM is conservatively estimated to account for approximately 2% of the total sugarcane mass, and therefore, approximately 37.4 Mt of PM are annually generated worldwide [82]. By-product wastes from the production of sugar from sugarcane include sugarcane trash, bagasse waste, ash, PM, and spent wash [83], of which bagasse and PM have higher economic values [84]. Currently, PM is used in soil fertilization, as a bio-sorbent, as animal feed, and as a source of chemicals, with a large amount of PM being landfilled [81, 85]; however, the application of PM to soil stabilization is relatively unexplored. Because the general compositions of PM are sugar, fiber, crude wax, crude protein, SiO_2_, CaO, P_2_O_5_, MgO, and total ash (Table 8) [86], it has the potential for use in soil stabilization. For example, fiber, as a tension-resisting material, could be used to reinforce soil to improve its shear strength, density, and compressibility [87, 88]. Notably, if the organic content of PM (fiber) exceeds a certain amount, it may be detrimental to stabilization [44]. Sugar indicates the presence of saccharides, which allow the material to function as a hydrocolloid [89] and form adhesive gels that act as connecting bridges between soil particles, thus increasing the cohesion and mechanical strength of the soil [90]. However, if the sugar is in the form of sucrose, it may reduce the strength development of cement-treated soil [91]. The pore filling effects caused by the presence of calcium carbonate (CaCO_3_) in PM may densify the soil matrix and enhance the soil strength. When PM is used as a partial lime replacement material, its high CaO content can provide additional calcium ions to increase electrolyte concentration and ion exchange capacity thereby decreasing the plasticity achieved by lime [85]. Notably, the presence of phosphorus pentoxide (P_2_O_5_) in PM is known to affect strength development [89].

**Table 8:** General composition of PM [86]

Composition	Mass fraction (%)
Sugar	5–15
Fiber	15–30
Crude wax	5–14
Crude protein	5–15
SiO_2_	4–10
CaO	1–4
MgO	0.5–1.5
P_2_O_5_	1–3
Total ash	9–10

James [85] investigated the potential of PM as a secondary material to improve lime-treated expansive soil. The results revealed that the benefits of adding PM to lime-treated expansive soil are pronounced in immediate and early strengths, suggesting its potential use as a strength accelerator in lime-treated soil. The strength enhancement in lime-treated expansive soil reaches peaks when the PM content is 0.25%. When the PM content exceeds 0.25%, the strength enhancement decreases due to the high organic content (15–30% in PM as reported by Partha and Sivasubramanian [86]). The addition of PM to lime-treated expansive soil modifies the mineral types formed during pozzolanic reactions, thus affecting the strength enhancement. Gumanta et al. [92] investigated the effect of PM as an alternative material for improving the performance of mudstone soil through a series of experimental tests including UCS, free swell, one-dimensional consolidation, and three-dimensional volumetric shrinkage tests. Besides, microstructural analyses were carried out to identify the mechanisms involved in forming SRS. Unlike other studies, in this study, PM was used alone to improve soil behavior. The results indicated that PM could act as a binder, effectively enhancing both the peak and postpeak strengths of mudstone soils. Additionally, treatment with PM improved swelling and shrinkage characteristics. However, the addition of PM also led to a slight increase in the compressibility of mudstone soils. It should be noted that no detrimental effects on mudstone soil stabilization were observed. Microstructural analyses revealed that the enhanced performance of mudstone soil with the addition of PM can be attributed to the formation of additional cementitious gels (C–S–H, C–A–H, and C–A–S–H gels). The studies on the role of PM for forming SRS are summarized in Table 9.

**Table 9:** Use of PM for forming SRS

Reference	Soil	Treatment	Treatment content	Tests	Effects of treatment	Primary mechanism	Remarks^a^
James and Pandian [89]	Expansive soil	PM; lime	0–2% (PM); 3% and 5.5% (lime)	UCS; Atterberg limits; pH	Increases UCS and decreases plasticity	Cementation of hydration products	The optimum PM content for strength improvement was 0.25%
James [85]	Expansive soil	PM; lime	0–2% (PM); 7% (lime)	UCS; Atterberg limits; XRD; X-ray Fluorescence (XRF); SEM; shrink-swell	Increases UCS and decreases plasticity at lower content of PM	Cementation of hydration products	The optimum PM content for strength improvement was 0.25%
Gumanta et al. [92]	Mudstone soil	PM	0–16%	UCS; free swell; 1D consolidation; 3D volumetric shrinkage; XRD; XRF; SEM; FT-IR	Increases UCS, strain at failure, and compressibility (slightly) and decreases swelling and shrinkage characteristic	Cementation of hydration products; Reinforcement	The level of improvement increases with an increase in additive content

^a^Under the studied conditions

### Municipal solid wastes used for forming SRS

Municipal solid waste refers to waste generated by households, offices, small-scale institutions, and commercial enterprises and managed by the municipality [3]. The studies related to waste tires and FM used for forming SRS are presented in the following sections.

#### Waste tire

Over the past decades, the increasing volume of waste tires and the difficulties associated with their proper disposal have led to worsening environmental problems. Approximately 263.4 million scrap tires were generated in the United State in 2019, approximately 15% of which are disposed of in landfills [93]. Therefore, identifying alternatives for the beneficial reuse of discarded tires represents an acute need. Due to unique properties, such as high durability, promising strength and compressibility, resiliency, and high frictional resistance, waste tires offer significant value in terms of reutilization in a wide variety of geotechnical engineering applications, including as lightweight fill and use in embankment construction [94, 95]. According to ASTM D6270-20 [96], scrap tires are classified into granulated tires (dimensions from 425 μm to 12 mm), tire chips (dimensions from 12 to 50 mm), and tire shreds (dimensions from 50 to 305 mm). Many studies have investigated the performance of diverse types of soils mixed with waste tires of dissimilar sizes, which have confirmed that waste tire has potential as an alternative material in geotechnical engineering applications [94, 95, 97–100].

Ghadr et al. [99] conducted a series of experimental tests, including consistency limit and linear shrinkage, UCS, free swell, and filter paper tests, to determine the optimum granulated tire content for use in expansive soil. The results showed that the addition of granulated tire to the expansive soil substantially decreased the swelling potential, modestly reduced the compression index, and lowered the bulk density. The inclusion of granulated tires resulted in a decrease in shear strength and stiffness, but an increase in failure strain, exhibiting a ductile postpeak plastic behavior. Expansive soil mixed with 15–20% granulated tire by dry weight appeared optimal for improving the soil properties. A series of consolidated drained (CD) triaxial compression tests were performed by Zornberg et al. [95] to evaluate the optimum content and aspect ratio (defined as the ratio of the length to width of an individual tire shred) of waste tire shreds in sands. The stress–strain and volumetric strain behaviors of the tire shred–sand mixture were affected by the tired shred content and aspect ratio. The optimum shred tire content, leading to the maximum shear strength, is approximately 35%. Under the same tire shred content, increasing the aspect ratio (from 1 to 8) of the tire shred led to an increase in overall shear strength. This increase may be attributed to the tensile force induced by the presence of tired shreds. Additionally, the effects of waste tires on the liquefaction resistance of silty sand have been studied by Ghadr et al. [94]. The waste tire has a relatively higher elasticity than sand; thus, it lends a damping effect to elements, relaxes skeletal stresses, and reduces the chance of sand particles' contact destruction, thereby further increasing the liquefaction resistance. The studies on waste tires used for forming SRS are summarized in Table 10.

**Table 10:** Use of waste tire for forming SRS

Reference	Soil	Treatment	Treatment content	Tests	Effects of treatment	Primary mechanism	Remarks^a^
Zornberg et al. [95]	Sand	Tire shred	0–100%	CD triaxial compression	Increases axial strain at failure and shear strength; shows dilatant behavior	Reinforcement	The optimum tire shred content was approximately 35%
Araujo et al. [97]	Lateritic soil	Tire shred	0–7.5%	Compaction; medium-scale direct shear	Decreases MDD; minimal variation in OMC	Reinforcement	The optimum tire shred content was 5%
Reddy et al. [100]	Sand	Tire chip	0–70%	Specific gravity; unit weight; large direct shear	Increases shear strength properties and decreases void ratio and dry unit weight; Improves compressibility characteristics and high load-carrying behavior	Reinforcement	The optimum tire chip content was in the range of 30–40%
Ghadr and Javan [98]	Sand	Tire shred	0–25%	Consolidated undrained triaxial compression	Increases axial strain and decreases shear strength, peak index, and secant and tangent Yang’s modulus	Reinforcement	The level of improvement increases with an increase in additive content
Ghadr et al. [94]	Silty sand	Tire shred	0–5%	Undrained cyclic triaxial compression	Increases liquefaction resistance	Reinforcement; higher elasticity of tire shred	The comparatively better cyclic performance of soil mixtures containing 2.5– 5% tire shred
Ghadr et al. [99]	Expansive soil	Granulated tire	0–100%	Consistency limit and linear shrinkage; UCS; free swell; filter paper	Increases failure strain and decreases swelling potential, compression index, bulk density, shear strength, and stiffness	Reinforcement	The optimum granulated tire content was in the range of 15–20%

^a^Under the studied conditions

#### FM

Due to the COVID-19 pandemic, the use of personal protective equipment, such as FM, has drastically increased worldwide. Approximately 129 billion FMs were being disposed of into the environment monthly as of June 2020 [101]. Although useful in preventing the spread of COVID-19, FMs end up in landfill or being incinerated. The increase in FM disposal has led to an increased demand for incinerators and landfill capacity, posing a threat to the environment and economy. Energy consumption and carbon production during incineration often exceed the amount stipulated in the carbon neutralization policy agreed upon by many countries. Meanwhile, FMs, which are nonbiodegradable, reportedly take hundreds of years to break down in landfills [87].

In general, the main components of the FM are nonwoven fabric, melt-blown nonwoven fabric, and polypropylene composite fiber [87]. Because of their flexibility, they have the potential to improve soil properties. Recently, some studies have investigated the potential of using FM for reinforcing soils [87, 102–104]. Ghadr et al. [87] explored the applicability of shredded FMs as an alternative for sand reinforcement by conducting a series of consolidated undrained (CU) triaxial compression tests. The experimental results revealed that using shredded FM as reinforcing material can considerably improve the undrained shear strength of the sands and produce dilative and strain-hardening behaviors. Such improvements increased with increasing FM length and decreasing D_50_ (diameter corresponding to 50% finer) of the sample. The strengthening effect of FM is likely due to the enhanced interface interaction between FM and sand. To evaluate the static and dynamic behaviors of granular soil reinforced with FM chips, Zhang et al. [102] conducted a series of monotonic and cyclic triaxial tests with various confining pressure and FM chip contents. The results indicated that the addition of FM chips to granular soil results in an increase in the shear strength and internal friction but a reduction in the stiffness, shear dilatancy, and delayed peak state under monotonic loading. Meanwhile, under cyclic loading, the energy absorption capacity increased significantly with the increase in FM chip content, and the settlement slightly increased; however, the resilience modulus decreased. The increase in strength caused by the addition of FM chips can be explained by the FM chip exerting additional tension on the surrounding particles to suppress their movement. The studies on FM used for forming SRS are summarized in Table 11.

**Table 11:** Use of FM for forming SRS

Reference	Soil	Treatment	Treatment content	Tests	Effects of treatment	Primary mechanism	Remarks^a^
Ghadr et al. [87]	Sea sand; Ottawa sand	Shredded FM	0–0.5%	CU triaxial compression; SEM	Increases undrained shear strength; produces dilative and strain-hardening behaviors	Reinforcement	The optimum FM contents were 0.3% and 0.5% for Ottawa sand and sea sand, respectively
Rehman and Khalid [104]	Fat clay	Shredded FM; silica fume	0–1.2% (FM); 0–16% (silica fume)	Compaction; UCS; 1D consolidation; CBR	Increases OMC, UCS (from 0–0.9% of FM), and CBR and decreases MDD	Reinforcement (FM); cementation of hydration products (silica fume)	The optimum FM contents was 0.9% for the considered silica fume content till 12%
Xu et al. [103]	Residual soil	FM chip	0–5%	CD triaxial compression; laser scanning microscopic (LSM); SEM	Increases peak shear strength (0.3–1% of FM) and elastic modulus (under limited amounts of FM)	Reinforcement	The optimum FM content was 0.5%
Zhang et al. [102]	Granular soil	FM chip	0–15%	Monotonic and cyclic triaxial	Increases shear strength, internal friction angle, energy absorption capacity, and settlement and decreases the stiffness, shear dilatancy, delayed peak state, and resilience modulus	Reinforcement	The level of improvement increases with an increase in additive content
Samadzadeh et al. [105]	Silty sand	Shredded FM	0–1%	Undrained cyclic triaxial shear	Dilative behavior; increases dissipation of excess pore water pressure, liquefaction resistance, shear modulus	Reinforcement	The level of improvement increases with an increase in additive content; the effectiveness of FM reinforcement diminished with increasing the median grain size (D_50_) of soil

^a^Under the studied conditions

## Other solid wastes used for forming SRS

In addition to the above-mentioned solid wastes, Table 12 summarizes some other solid wastes used for forming SRS, including lime kiln dust (LKD), calcium carbide residue (CCR), recycled bassanite, and bagasse ash, with a brief description of their source, composition, and soil improvement mechanism.

**Table 12:** Summary of some other solid wastes used for forming SRS

Solid waste	Source	Main composition	Primary mechanism
LKD	A by-product of the manufacture of quick lime	A high amount of calcium	LKD is usually combined with FA for soil stabilization. The active calcium in LKD complements Al_2_O_3_ and SiO_2_ in FA to form C–A–S–H gel
CCR	A by-product of acetylene, polyvinyl chloride, and polyvinyl alcohol production process	Calcium hydroxide	In the presence of pozzolanic materials (FA, GGBS, and RHA), it provides cementitious function
Recycled bassanite	Production from the gypsum waste	CaSO_4_ $\bullet$ 1/2H_2_O	Recycled bassanite is a soluble material, so it is usually mixed with cement or lime as a solidification agent. Formation of ettringite and other cementitious gels
Bagasse ash	A by-product of the combustion process of bagasse, which is the residue obtained by the sugar industry after extracting the juice from sugarcane	Amorphous silica	As a pozzolanic material

## Discussion

### Mechanisms behind SRS

From the introduction of waste management as applied in soil stabilization, several mechanisms facilitate the use of solid waste for forming SRS, including pore filling, cementation of hydration products, and reinforcement. A summary of these mechanisms is illustrated in Table 13.

**Table 13:**
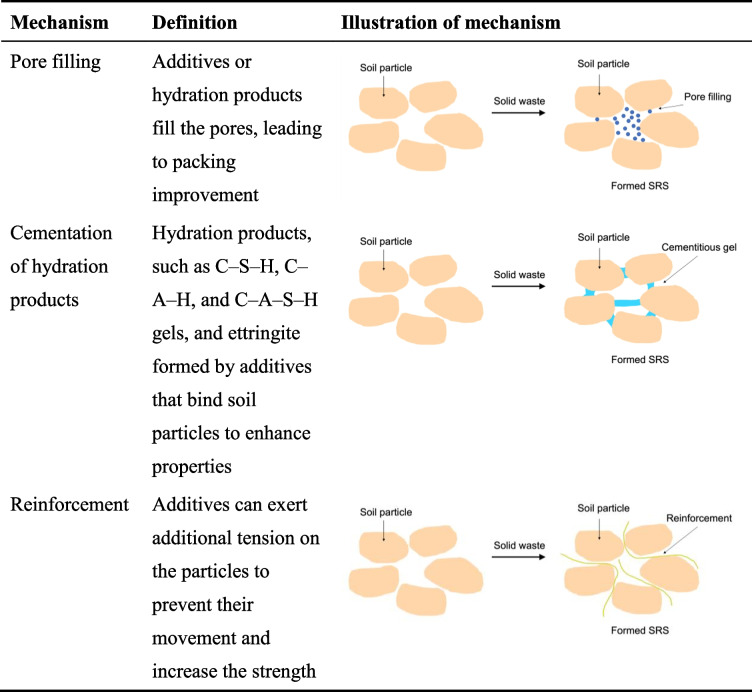
Summary of mechanisms behind SRS

### Discussion on potential toxic elements present in solid wastes

In recent years, environmental pollution by potential toxic elements in waste has garnered increasing attention. Potential toxic elements refer to chemical elements, including metals and nonmetals that, in high concentrations, can cause potentially harmful effects on organisms [106]. Metal(loid)s, such as chromium, arsenic, selenium, cadmium, mercury, and lead, are extensively employed in various industrial processes, posing a significant challenge in the management of solid waste due to their potential acute and chronic hazards to both human health and ecosystems [106–108]. Therefore, it is essential to consider the potential toxicity of solid waste generated from industrial processes (such as industrial solid waste and municipal solid waste). Table 14 lists the potential toxic substances present in the industrial and municipal solid waste discussed in this study. It is important to note that while the high alkalinity of RM makes its disposal a major challenge and poses serious environmental issues, such as groundwater pollution, it can also be utilized as an environmental remediation material for wastewater purification, waste gas purification, and soil remediation [49, 109]. As an aqueous solution adsorbent, RM can effectively remove various pollutants, including inorganic anions such as phosphate, fluoride, ferricyanide, boron, and nitrate, heavy metals like As, Cr, Pb, and Cu, as well as organic pollutants [109]. Moreover, it has been reported that alkali-activated GGBS exhibits superior capability for immobilizing heavy metals [110]. The treatment of waste with waste is an efficient method for environmental protection as it allows for the simultaneous treatment of multiple wastes, resulting in a positive outcome.

**Table 14:** Potential toxic substances present in the industrial and municipal solid wastes

Solid waste	Potential toxic substances	Reference
FA	Heavy metals: Cr, Cu, Mn, Ni, and Zn	[111]
RM	Alkali	[112]
C&D waste	Heavy metals: Cd, Cr, Cu, Zn, Pb, and As	[113]
Waste tire	Polycyclic aromatic hydrocarbons, heavy metals (Pb), phthalates, volatile organic hydrocarbons and other semi-volatile organic hydrocarbons	[114]
FM	polychlorinated dibenzo-para-dioxin and polychlorinated dibenzofuran	[115]

A major concern in utilizing solid waste generated from industrial processes is the leachate generated, which may pollute groundwater beyond the allowable limits set by the World Health Organization and US EPA. Few studies have examined metal leaching from mixtures of soil and solid waste generated from industrial processes in recent years [116–118]. For example, Goswami and Mahanta [116] investigated the leaching characteristic of heavy metals from residual lateritic soil stabilized with FA and lime through a single batch leaching test and column leaching tests. Their results revealed that the high pH induced by lime addition helps to retain most of the metals within the treated soil matrix.

Additionally, many researchers have investigated the treatment for reducing leaching [119–121], among which the most common and effective method is the use of various additives to immobilize the hazardous element present in solid waste generated from industrial processes. The main purpose of this method is to minimize the solubility, leaching, and toxicity of the contaminants. Importantly, Ghadr et al. [122] also emphasized the significance of containment techniques for the SRS and suggested that a new technique and precautions should be taken for field-scale applications.

## Summary and future work

This review presents an overview of the common and diverse solid wastes that have been studied or used for SRS. The conclusion can be drawn as follows:

- 1. Various types of solid waste are effective in improving the mechanical and hydromechanical behaviors of soils. The mechanisms behind SRS depend on the characteristics of both the soil and solid wastes.
- 2. The mechanisms behind soil reinforcement and stabilization using solid wastes are (a) pore filling, (b) cementation of hydration products, and (c) reinforcement.
- 3. The presence of activators can improve the reactivity of some solid wastes (e.g., FA, GGBS, and RHA).

Potential toxic elements from solid waste can be harmful to organisms in the environment. The use of various additives is an effective method of immobilizing the hazardous element present in solid waste generated from industrial processes.

Based on the identified gaps, some potential for future work is suggested below:

- 1. Adding some solid wastes (e.g., FA, RHA) to the soil significantly improves the UCS of the soil. However, treated soils may exhibit brittle behavior [74, 123]. Using waste tire or FM, tension-resisting materials, in FA- or RHA-treated soils can not only improve the soil ductility but also recycle more solid waste, further minimizing environmental pollution.
- 2. The literature review provides valuable insights into the mechanical and hydromechanical improvement of soil treated with solid waste. Sooner than later, numerical calibrations of the experimental results should be attempted to characterize the constitutive behavior of treated soils and a develop method to incorporate this behavior into the framework needed to numerically predict the response of treated soil. Also, the role of 3D printing techniques in SRS could become even more important.
- 3. The previous studies on the effects and mechanisms of various solid wastes to improve the hydromechanical and mechanical behaviors of soils have been conducted mainly through laboratory tests; however, model, large-scale, and field testing may be required to ensure practical applications. Additionally, the inclusion of field or large-scale tests with numerical simulations (adopting or developing more advanced constitutive models) would allow a deeper understanding of the effects of various solid wastes on SRS. Note that three-dimensional and seismic effects can be important factors affecting the performance of the SRS [124, 125] and should be carefully considered. In addition, the containment technique and potential impact of climate change on SRS (i.e., thermo-hydro-mechanical behavior) should be taken into account in the near future.

## Data Availability

This study does not generate new data and/or new computer codes.
